# Autonomic Substrates of the Response to Pups in Male Prairie Voles

**DOI:** 10.1371/journal.pone.0069965

**Published:** 2013-08-05

**Authors:** William M. Kenkel, Jamespaul Paredes, Gregory F. Lewis, Jason R. Yee, Hossein Pournajafi-Nazarloo, Angela J. Grippo, Stephen W. Porges, C. Sue Carter

**Affiliations:** 1 Brain and Body Center, Department of Psychiatry, University of Illinois at Chicago, Chicago, Illinois, United States of America; 2 Division of Psychobiology, Yerkes National Primate Research Center, Emory University, Atlanta, Georgia, United States of America; 3 Department of Psychology, Northern Illinois University, DeKalb, Illinois, United States of America; 4 Department of Psychiatry, University of North Carolina, Chapel Hill, North Carolina, United States of America; 5 Department of Psychology, Norteastern University, Boston, Massachusetts, United States of America; Tulane University Medical School, United States of America

## Abstract

Caregiving by nonparents (alloparenting) and fathers is a defining aspect of human social behavior, yet this phenomenon is rare among mammals. Male prairie voles (*Microtus ochrogaster*) spontaneously exhibit high levels of alloparental care, even in the absence of reproductive experience. In previous studies, exposure to a pup was selectively associated with increased activity in oxytocin and vasopressin neurons along with decreased plasma corticosterone. In the present study, physiological, pharmacological and neuroanatomical methods were used to explore the autonomic and behavioral consequences of exposing male prairie voles to a pup. Reproductively naïve, adult male prairie voles were implanted with radiotransmitters used for recording ECG, temperature and activity. Males responded with a sustained increase in heart-rate during pup exposure. This prolonged increase in heart rate was not explained by novelty, locomotion or thermoregulation. Although heart rate was elevated during pup exposure, respiratory sinus arrhythmia (RSA) did not differ between these males and males exposed to control stimuli indicating that vagal inhibition of the heart was maintained. Blockade of beta-adrenergic receptors with atenolol abolished the pup-induced heart rate increase, implicating sympathetic activity in the pup-induced increase in heart rate. Blockade of vagal input to the heart delayed the males’ approach to the pup. Increased activity in brainstem autonomic regulatory nuclei was also observed in males exposed to pups. Together, these findings suggest that exposure to a pup activates both vagal and sympathetic systems. This unique physiological state (i.e. increased sympathetic excitation of the heart, while maintaining some vagal cardiac tone) associated with male caregiving behavior may allow males to both nurture and protect infants.

## Introduction

Caregiving directed toward infants by nonparents, sometimes called alloparenting, is common to many human cultures [1] and is critical to human evolution and development [2]–[4]. Whereas the neurobiology of female parental behavior has received extensive attention [5], [6], male parental behaviors and alloparenting remain poorly understood. Understanding alloparental care is critically important for many reasons including the fact that children are between eight [7] and fifty [8] times more likely to suffer fatal abuse when living with an unrelated adult male, i.e. while under alloparental care.

Prairie voles (*Microtus ochrogaster*) are small, socially monogamous rodents that have been useful for understanding the neurobiological basis of various social behaviors [9], [10]. Male prairie voles display high levels of alloparental behavior [11] and often remain with their natal family, serving as alloparents [12].

Factors that may influence the expression of alloparental care include the physiological and emotional states of the alloparent. For example, stressful experiences, such as a forced swim or treatment with exogenous corticosterone, facilitate subsequent alloparental behavior in male, but not female prairie voles [13]. Our studies of the immediate consequences of pup exposure revealed a decreased concentration of plasma corticosterone compared to control subjects [14], initially suggesting the hypothesis that interactions with the pup might have anxiolytic properties; that is, males may approach and huddle over the pup as a means to cope emotionally with negative experiences.

Based on the above observations, we originally hypothesized that males’ alloparental interactions with a pup would be associated with a reduction in arousal. Furthermore, we hypothesized that this might be due to an increase in the vagal influences on the heart, manifesting in slower heart rate, with a concomitant increase in respiratory sinus arrhythmia (RSA). RSA is a sensitive measure of the component of cardiac vagal tone mediated through the myelinated vagal efferent pathways originating in the nucleus ambiguus, which allows an integrated social engagement system and permits a functional coupling of social behavior and emotion regulation [15], [16].

The dynamic regulation of heart rate is coordinated in several brainstem nuclei, including the nucleus ambiguus (NA), dorsal motor nucleus of the vagus (DMX), nucleus tractus solitarius (NTS), and rostral ventrolateral medulla (RVLM). The purpose of the present study was to use the male prairie vole as a model to examine the autonomic substrates of alloparenting. Using radiotelemetry, pharmacology and immunohistochemistry, we evaluated the changes in the ANS following exposure to a pup or to other social or nonsocial stimuli.

## Methods

### Animals

Male F2 or F3 descendants of prairie voles originally captured near Champaign, Illinois were used in these experiments. All of the experiments used sexually naïve adult males, 60–100 days old. Unrelated and unfamiliar pups, 1–3 days of age, as well as unrelated and unfamiliar adult females were used as stimuli.

Subjects were maintained on a 14 h/10 h light/dark cycle on at 06.30 h in a temperature and humidity controlled vivarium. Food (Purina rabbit chow) and water were available *ad libitum*. Prairie vole offspring remained in their natal group with their parents in large polycarbonate cages (24×46×15 cm) containing cotton nesting material. Offspring were weaned at 21 days of age, prior to the arrival of the next litter to prevent premature exposure to pups. After weaning, they were pair-housed with a same-sex sibling in smaller cages (17.5×28×12 cm) in a single-sex colony room until testing. Thus, all test subjects were sexually naïve and had never been exposed to pups. All procedures were conducted in accordance with the National Institutes of Health Guide for the Care and Use of Laboratory Animals and were approved by the University of Illinois at Chicago Institutional Animal Care and Use Committee. Experiments began during the lights-on period between 9∶00 h and 10∶00 h.

### Alloparenting Test and Behavioral Analysis

Following recovery from radiotransmitter implantation (Experiments 1, 2, 3, 4 and 5), baseline data were recorded from subject males while they remained with their brothers in the home cage for 30 minutes. Five minutes of stable, stationary data were averaged to obtain a baseline value for each animal. The siblings were removed and the subjects were immediately presented with one of three stimuli. Adult males in Experiments 6 and 7, were not implanted with radiotransmitters, but were transferred to new cages individually where behavioral testing was conducted and video-recorded for later analysis. Males were considered alloparental if they huddled over the stimulus pup [14], [17], [18]. In the rare instances in which males displayed aggression towards the pup, the test was immediately aborted and the health of the pup was assessed. Uninjured pups were returned to their parents and injured pups were euthanized. Videotapes of behavior were scored by two trained, experimentally blind observers (inter-rater reliability >95%, Noldus Observer, Noldus Inc.). Behavioral domains quantified included: first contact with the stimulus (latency), stimulus retrievals (frequency), licking and/or grooming the stimulus (duration), auto-grooming (duration), huddling over the stimulus (duration) that likely serves to protect the pups and regulate their body temperature, contact with the stimulus (duration) defined as the subject having contact with the stimulus but not huddling over it or licking and/or grooming it (duration) and “other” behaviors, defined as all other behaviors that were not directed at the stimulus (e.g. drinking, cage exploration).

### Radiotelemetry Recording Equipment Implantation

Males were implanted with wireless radiotelemetry transmitters [Data Sciences International (DSI), St. Paul, MN] according to procedures described previously [19]. Briefly, telemetric transmitters were implanted intraperitoneally under aseptic conditions, following anesthesia with ketamine (67 mg/kg sc; NLS Animal Health, Owings Mills, MD) and xylazine (13.33 mg/kg sc; NLS Animal Health, Owings Mills, MD). Animals were kept under a warming lamp, and the surgical area was shaved and cleaned before any incisions were made. Rostral-to-caudal skin and muscle incisions were made on the ventral surface of the abdomen. The transmitter was inserted into the abdominal cavity, then sutured to the muscle. The process of suturing the transmitter to the muscle thereby closed the incision. The leads from the transmitter were pulled rostrally using a trochar and sleeve under the skin, and anchored in place with permanent sutures (DII placement). Skin incisions were sutured closed, and subcutaneous fluids and analgesia (Carprofen) were administered as necessary. All animals were housed for 5 days in custom-designed cages (24×46×15 cm) after surgery [20]. These cages included a divider to permit adequate healing of suture wounds in the instrumented animal without socially isolating subjects from their siblings during recovery. Within these partitioned cages, animals are able to exchange visual, olfactory and auditory stimuli. Animals were then returned to the standard home cages (with their sibling) for an additional 5–7 days before the onset of experiments. In addition to their use during the subject’s recovery from surgery, these partioned cages also were utilized during Experiment 4.

### Radiotelemetric Recordings

Electrocradiogram (ECG), temperature and activity signals were recorded with a radiotelemetry receiver (DSI; sampling rate 5 kHz for ECG and 256 Hz for activity, 12-bit precision digitizing). This system allows for the recording of heart rate and derived measures of heart rate variability along with temperature and locomotor activity. Radiotelemetric data were quantified according to procedures previously described [20].

Heart rate was evaluated by visual inspection and using vendor software (Data Sciences International, St. Paul, MN), and R-wave detections were verified by visual inspection and with a custom-designed software package (CardioEdit 1.5, Brain Body Center, University of Illinois at Chicago). The R-R intervals were analyzed for variations (heart rate variability) using a custom-designed software package, that calculates amplitude of RSA [21].

RSA was assessed using standardized procedures [22]–[24], which were modified for the frequency band of spontaneous breathing the vole and described in detail elsewhere [19], [20], [25]. RSA was operationally defined as the natural log of the sum of the power within the respiratory bandwidth of 1.0–4.0 Hz. This procedure has been validated pharmacologically in prairie voles [20] and provides the greatest sensitivity to changes in vagal activity [21]. The amplitude of RSA represents the functional vagal impact on the sino-atrial node of myelinated vagal efferent pathways originating in the brainstem (NA). The ECG signal was exported into a data file and examined using a custom-designed software package (CardioEdit; Brain-Body Center, UIC) to ensure that all R waves were properly detected. The following procedures were implemented to minimize the possibility of violating the assumption of stationarity which can distort time series analyses of RSA: 1) the R–R intervals (heart period) were time-sampled into equal time intervals with a sampling rate of 20 Hz, 2) the time series were detrended with a moving polynomial filter that removed variance in the series below 1 Hz for RSA (i.e., 21-point cubic polynomial), 3) time series analyses of the detrended data quantified the variance within the frequency band of spontaneous breathing as a measure of RSA, and 4) the derived variances representing RSA were logarithmically transformed. e spectral analyses identified the peak amplitude of RSA.

### Tissue Fixation

A spinning immersion fixation protocol [26] was used to preserve brain tissue for immunohistochemistry in Experiment 7. Brains were carefully extracted from the skull and placed in an ice-chilled scintillation vial containing 19 ml of 4% buffered paraformaldehyde and 1 ml of 5% acrolein and spun at low speed for 10 minutes. Brains were then blocked, exposing the lateral ventricles and returned to the fixative solution for an additional 1 hour and 50 minutes. Brains were then placed in a fresh fixative solution and spun for an additional 2 hours. Subsequently, brains were immersed in a 25% sucrose solution and stored at 4°C until sectioned. Tissue containing brainstem autonomic regions of interest were cut in 40 µm coronal plane sections using a freezing sliding microtome. Sections were stored in cryoprotectant at −20°C until processed.

### Immunohistochemistry

Brains were stained for c-Fos using standard avidin-biotinylated enzyme complex (ABC) immunocytochemistry (Vector Labs; Burlingame, CA). Serial sets (every 3^rd^ section) of free-floating tissue sections were rinsed in 0.05 M potassium phosphate buffered saline (KPBS) to remove excess cryoprotectant. Sections next were incubated in 1% sodium borohydride for 20 minutes at room temperature (RT) to reduce free aldehydes to alcohol followed by a rinse in KPBS. Sections then were incubated for 15 minutes in 0.014% phenylhydrazine at RT to block endogenous peroxidase activity and rinsed again in KPBS. Next, sections were incubated in rabbit c-Fos antisera (Santa Cruz Biotechnology Inc., Santa Cruz, CA) at 1∶50,000 concentration in 0.05 M KPBS +0.4% Triton X-100 for 1 hour at RT and for an additional 48 hours at 4°C. Sections were rinsed in KPBS before being incubated for 1 hour at RT in biotinylated goat, anti-rabbit IgG (Vector Labs; 1∶600 dilution in KPBS +0.4% Triton X-100). Sections were rinsed again in KPBS and then incubated in an avidin-biotin peroxidase complex (45 µl A, 45 µl B per 10 ml KPBS +0.4% Triton X-100; Vectastain ABC kit-elite pk-6100 standard; Vector Labs) for 1 hours at RT. Sections were rinsed in KPBS and then with tris buffered saline. c-Fos immunoreactivity (ir) was visualized following incubation in a solution containing 50 ml of tris buffered saline, 1.25 g nickel sulfate, 41.5 µl of 3% H_2_O_2_ and 10 mg of diaminobenzidine for 15 minutes at RT. Sections were rinsed in sodium acetate followed by a series of rinses in KPBS.

Labeled sections were mounted on gelatin-coated slides and air-dried overnight. Sections then were dehydrated in ascending ethanol dilutions and cleared with Histoclear (National Diagnostics). Slides were cover slipped with Histomount (National Diagnostics).

### Quantification of Immunoreactivity

Slides were coded and images of hypothalamic sections were acquired using a Nikon Eclipse E 800 microscope, Sensi-cam camera, and IP Lab 3.7 computer software (Scanalytics Inc., Fairfax, VA). Images were captured using a 4X microscope objective and scored using ImageJ (NIH software). c-Fos-immunoreactivity (ir) was quantified relative to background staining, which was minimal, and was measured if staining exceeded an arbitrary threshold the intensity of background. In 8-bit grayscale, threshold was set at 120, with background staining on average approximately 210+/−20. As a stain, c-Fos appears as small punctate areas corresponding to cell nuclei. Quantification was limited to specific labeling by excluding labeling that was outside the size range of nuclei using both automatic and manual inspection. Optical density was quantified bilaterally using standardized sampling areas in sections matched to the mouse brain atlas, Figure 93 [27]. Three brain sections were analyzed from each subject and results were averaged bilaterally across sections. Caudal sections of each brain region were chosen to better guarantee that these neurons would represent cardiovascular control regions [28], [29]. The location of the NA in the vole was confirmed by staining a subset of slices with choline acetyltransferase [30], generously provided by Dr. David Wirtshafter.

### Experimental Design

The present study consisted of 8 experiments. Experiment 1 was designed to examine the heart rate and RSA responses of the naïve male to pup exposure. To address the question of whether changes in heart rate and RSA were due to stimulus novelty, Experiment 2 examined the effect of repeated pup exposures while Experiment 3 observed heart rate and RSA during an extended, hour-long period of pup exposure. Experiment 4 evaluated whether or not direct physical contact was required to produce the heart rate increase. To do this, we used our custom designed cages that enable the pup to be partitioned from the adult male by means of a barrier. The barrier prevented males from touching the pup, but did not limit visual, auditory and olfactory stimulation. Experiment 5 evaluated the sympathetic role in autonomic and behavioral responses by administering a β adrenergic antagonist (atenolol). Experiment 6 evaluated the vagal influences on the behavioral response to a pup by administering a muscarinic acetylcholine antagonist (atropine) prior to pup exposure. Finally, Experiment 7 used c-Fos immunohistochemistry to examine the effects of pup exposure on the activity of autonomic brainstem nuclei.

### Experiment 1 (Pup Exposure)

Adult males (n = 12), implanted with radiotransmitters, were presented sequentially with three stimuli: an unrelated pup (1–3 days old), a pup-sized wooden dowel, or an unrelated, unfamiliar adult female (60–90 days old). The order of the pup and dowel conditions was randomized. Exposure to the adult female always came last, because exposure to a member of the opposite sex can activate reproductive processes in this species, which in turn might have affected the response to a pup. Males were exposed to each of the three conditions and each exposure was separated by one week. Female prairie voles do not undergo spontaneous estrus and thus were not sexually receptive during these exposures. Aggressive displays often were observed between the male and the stimulus female upon exposure; however, these interactions were non-violent and did not result in injury. Stimulus females were used only once each. All stimulus presentations lasted for 20 minutes during which time ECG, temperature, and activity were recorded. ECG data were then analyzed for heart rate and RSA.

### Experiment 2 (Repeated Pup Exposures)

Radiotransmitters were implanted in a second cohort of animals (n = 7) and behavior was recorded as previously described. These subjects were exposed to a pup, following the procedures described in Experiment 1; however, pup exposure was repeated on three separate occasions at 2 day intervals to examine possible effects of novelty and to determine whether heart rate and RSA responses changed in response to repeated pup exposure. Only pups of 1–3 days of were used and males were exposed to a novel pup on all 3 occasions.

### Experiment 3 (Hour-long Pup Exposure)

A third cohort of animals (n = 7) was implanted with radiotransmitters and behavior was recorded as previously described. In contrast to Experiments 1 and 2, this cohort was exposed to either a pup or a dowel for 60 minutes to determine whether heart rate and RSA responses habituated after a longer exposure period. The order of exposure to pup or dowel was randomized and tests were separated by 2 days.

### Experiment 4 (Divided Cage)

A fourth cohort of animals (n = 7) was implanted with radiotransmitters and behavior was recorded as described for the 3 previous experiments. In contrast to those experiments, these subjects and their siblings were transferred to large cages (24×46×15 cm) 24 h prior to testing. These cages were similar to those used during the animals’ recovery from surgery. The subject and sibling remained in the new large cage without a cage divider until the time of testing. The sibling was removed from the cage immediately prior to testing. After removal of the sibling, the cage dividers were reinstalled and the subjects in this cohort were presented with a pup in the middle of the chamber across the barrier. These custom-designed cages allowed males to be exposed to various pup stimuli (e.g. vocalizations, visual and olfactory stimuli) while preventing them from interacting directly with the pup. To compare conditions within subject, the divider was removed after 10 minutes, permitting direct interactions with the pups for an additional 10 minutes, after which time the pup was removed and the testing ended.

### Experiment 5 (Atenolol Pretreatment)

A fifth cohort of animals (n = 6) was implanted with radiotransmitters and behavior was recorded as previously described. In this cohort, adult males received an intraperitoneal injection of either atenolol (Sigma-Aldrich, St. Louis, MO; 8 mg/kg) or saline vehicle 30 minutes prior to the introduction of the pup. The pup was placed in the cage for 20 minutes. Animals received both conditions in a counter-balanced order, spaced 2 days apart and different pups were used on each occasion. The timing and dosage were based on our previous work in this species [20]. Atenolol is a selective β_1_ receptor antagonist which blunts the peripheral sympathetic nervous system’s ability to increase heart rate; atenolol does not cross the blood-brain barrier.

### Experiment 6 (Atropine Pretreatment)

Subjects (n = 22) in Experiment 6 were not implanted with telemetry devices. These animals were randomly assigned to one of two condition groups, either receiving an intraperitoneal injection of atropine methyl nitrate (i.e. atropine, 4 mg/kg; Sigma-Aldrich, St. Louis, MO; n = 12) or saline vehicle (n = 10) 30 minutes prior to the onset of a 20 minute pup exposure. The timing and dosage used were based on previous work research in voles, in which heart rate was monitored [20], and based on the consistent autonomic changes seen in that study, telemetry was not used here. Behavior towards the pup was compared as a function of atropine versus vehicle (saline). Atropine is a competitive antagonist for the muscarinic acetylcholine receptor and blocks parasympathetic input to the heart without crossing the blood-brain barrier.

### Experiment 7 (Immunohistochemistry)

A seventh cohort of animals, not implanted with a radiotransmitter, was used to examine the effects of pup exposure versus control procedures on brainstem nuclei that have been implicated in the regulation of the autonomic nervous system. Adult male voles were randomly assigned to two condition groups: Pup (n = 30) or Dowel (n = 22). Hypothalamic data from these animals were previously published in a separate study [14]. Following a 20 minute alloparental test, animals remained in the testing cage for an additional 40 minutes, and then were sacrificed. This timing was selected to maximize the expression of c-Fos in response to the stimuli presented. Tissue was collected and processed for immunohistochemistry in brainstem autonomic regions (i.e. DMX, NA, NTS and RVLM).

### Statistical Analysis

The data are presented as means ± standard errors in all figures. In animals instrumented for telemetry, heart period was extracted from the ECG data and were quantified (i.e., msec between successive R-waves). Heart period data have been transformed to heart rate (beats per minute) in the figures to facilitate visual interpretation. Heart period, RSA, activity, and temperature data were analyzed using repeated measures analyses of variance (ANOVA) with time and stimulus/condition as within subject factors. The correlation between heart rate and RSA was calculated with a Pearson’s correlation and the strength of the correlations was compared between conditions via ANOVA after Fisher transformation. Alloparental behavior was assessed in Experiments 2–6. The latency to approach the pup, the amount of time in contact with the pup, the amount of time licking/grooming and the amount of time huddling over the pup were compared between conditions (including drugs, repeated exposures, and so forth) by means of a two tailed paired samples t-test (Experiments 2 and 3) or one tailed t-tests based on an *a priori* hypothesis that: (Experiment 5) responsiveness to the pup would be positively affected by atenolol and (Experiment 6) atropine would impair these behaviors. In Experiment 7, one tailed t-tests were used to compare the pup and dowel conditions based on *a priori* hypotheses that pup exposure would induce increased activity in the NA, NTS and DMX. All statistical analyses were conducted using SPSS 19.0 with α set at 0.05.

## Results

### Experiment 1 (Pup Exposure)

**Figure 1:**
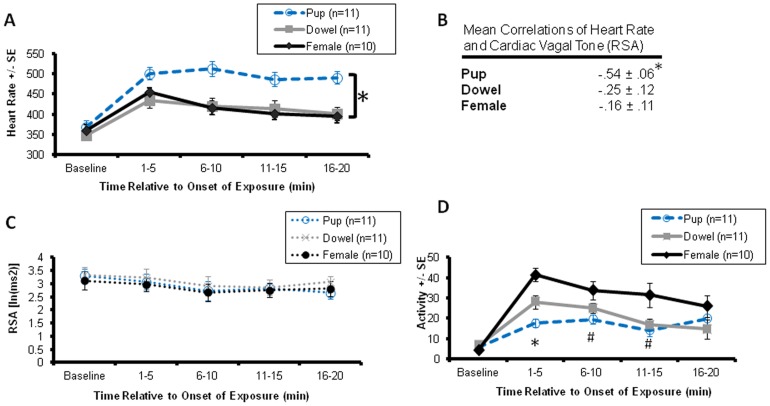
Respiratory sinus arrhythmia (RSA) is maintained and heart rate increased during alloparental behavior which is not explained by locomotor activity. (**A**) Heart rate during exposure to social (pup, female) and non-social (dowel) stimuli yielded main effects of stimulus and time, with the pup condition generally highest. (**B**) The correlation between heart rate and RSA during exposure was highest in the pup condition. (**C**) There were no effects on RSA other than a main effect of time. (**D**) Locomotor activity yielded main effects of stimulus and time as well as an interaction between stimulus and time. * indicates the effect of stimulus, such that both the dowel and female stimuli were significantly different than the pup condition (p<0.05), # difference significant only between the female and pup groups (p<0.05).

The results from Experiment 1 are shown in Figure 1. Data collected from one animal was excluded from Experiment 1 due to poor signal quality. All males expressed alloparental behavior during these exposures (n = 11). The signal quality deteriorated in another subject just prior to the female test condition, so this subject also was excluded from analysis. A repeated measures ANOVA of binned heart rate yielded a main effect of stimulus [F(2,8) = 15.72, p = 0.002] and time [F(4,6) = 32.7, p<0.001], with the pup condition producing higher heart rate than both the dowel and female stimuli (p = 0.001 for both comparisons, Fig. 1A). Heart rate increased at the beginning of the exposure and then gradually returned to baseline. Across the 20 minutes of stimulus exposure, the correlations between heart rate and RSA were significantly stronger in the pup condition (–.54±.06), compared to either the dowel condition (–.25±.12) or the female condition (–.16±.11, p<0.02 for both comparisons, Fig. 1B).

RSA did not differ during exposure to a pup, dowel or a female; however, there was a main effect of time on RSA [F(4,6) = 9.56, p = 0.009]. RSA gradually decreased with time during all 3 conditions (Fig. 1C). Analysis of the binned activity data yielded main effects of stimulus [F(2,8) = 8.23, p = 0.011] and time [F(2,8) = 86.97, p<0.001] as well as an interaction effect between stimulus and time [F(2,8) = 181.23, p = 0.005, Fig. 1D]. Relative to baseline, both dowel and female conditions increased activity significantly more than the pup condition at the beginning of the exposure (p≤0.011 for these comparisons). The female condition was significantly more active than the pup (female = 27.451±3.850 counts, pup = 14.468±1.908 counts, p = 0.003); the dowel condition was intermediate and did not differ from either female or pup. No group differences were found for temperature.

### Experiment 2 (Repeated Pup Exposures)

**Figure 2:**
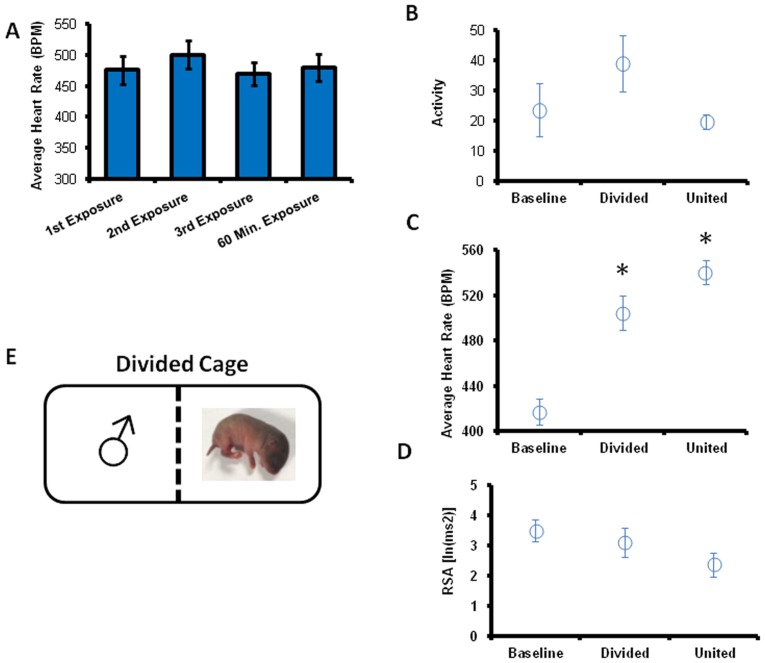
The cardiovascular response to a pup does not readily habituate. (**A**) Heart rate during interaction with a pup did not differ during repeated pup exposures (1st three bars) or during prolonged pup exposure (4th bar) (p>0.05). Time was collapsed across the 10 minutes of each condition (baseline, divided cage, united with pup) to yield a main effect of condition on heart rate (**B**), no effect on activity (**C**) and a trend towards an effect on RSA (**D**) (p>0.05). * indicates p<0.05 in comparison to both other conditions.

The heart rate results of repeated pup exposures are shown Experiment 3 in Fig. 2A. All males expressed alloparental behavior towards the pup during each of the three testing sessions (n = 7). Differences in heart period, RSA, temperature, or activity were not observed between the 1^st^, 2^nd^, and 3^rd^ pup exposures. The strength of the correlation between heart rate and RSA also did not differ across exposures (p>.05). However, behavior did not differ in any measured domain between any of the exposures (p>.05).

### Experiment 3 (Hour-long Pup Exposure)

Figure 2A shows the cardiovascular results of hour-long pup exposure. All males expressed alloparental behavior towards the pup during each of the three testing sessions (n = 7). No differences were found between the first 10 minutes and the last 10 minutes during the hour-long pup exposure in terms of: heart period, RSA, temperature, activity, or the strength of the correlation between heart rate and RSA (all p>.05). Behavior did not differ in any measured domain between any of the exposures (p>.05).

### Experiment 4 (Divided Cage)

Figure 2B–E shows the results of pup exposure within a divided cage. All males expressed alloparental behavior towards the pup (n = 7). Males and pups began Experiment 4 physically separated by a barrier. When a pup was on the other side of the barrier, male voles spent most of their time investigating the barrier and digging at the base of the barrier (88±4% combined). Following the removal of the barrier, males responded to the pup with alloparental care that did not differ from behavior observed in other experiments (p>0.05). Across the three conditions (baseline, pup across barrier and barrier removed), there was a main effect of condition on heart rate [F(2,5) = 38.88, p = 0.001]. There was also a trend towards a decrease in RSA (p = 0.051) and no effect on activity. Compared to baseline, when the pup was across the barrier, heart rate was higher when the pup was placed in the cage on the opposite side of the barrier (p = 0.003). Heart rate remained elevated after the barrier was removed and the subject was allowed to interact with the stimulus pup. Heart rate was higher in males allowed access to the pup compared to both baseline (p<0.001) and during the time while the pup was on the other side of the barrier (p = 0.035). Temperature and the strength of the correlation between heart rate and RSA were not affected by testing conditions (p>.05).

### Experiment 5 (Atenolol Pretreatment)

**Figure 3:**
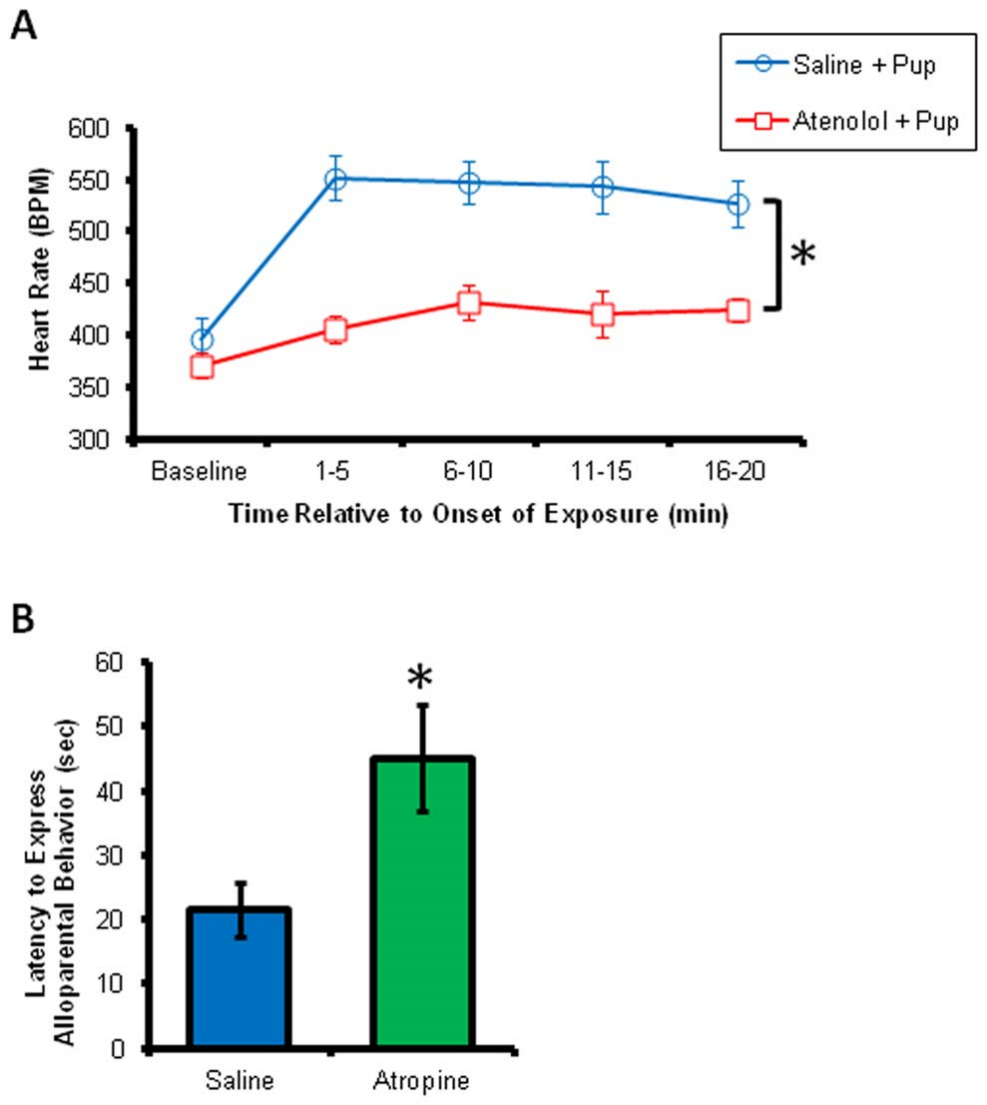
Pharmacological manipulations illustrate the contributions of the sympathetic and parasympathetic branches of the autonomic nervous system. (**A**) Atenolol (8 mg/kg, i.p.) blocked the pup-induced heart rate increase, with main effects of time and treatment. * indicates the effect of treatment, such that the saline treatment produced a higher heart rate than the atenolol treatment. (**B**) Atropine (4 mg/kg, i.p.) delayed initial approach to the pup. * indicates p<0.05.

Figure 3A shows the results of atenolol pretreatment. All males in Experiment 6 expressed alloparental behavior when presented with a pup following both the saline and atenolol injections (n = 6). Atenolol pretreatment had no effect on baseline heart rate, activity or on either the quantity or quality of alloparental behavior (p>0.05). In contrast, a repeated measures ANOVA assessing heart rate during pup exposure detected main effects of time [F(4,2) = 676.59, p = 0.001] and condition [F(1,5) = 8.28, p = 0.035]. Average heart rate in the atenolol condition was lower than during the saline condition (p<0.05). There were no effects of atenolol on temperature or the strength of the correlation between heart rate and RSA (p>.05).

### Experiment 6 (Atropine Pretreatment)

Figure 3B shows the results of atropine pretreatment. Atropine did not affect the overall tendency to express alloparental behavior as 10 of 12 atropine-treated animals and 7 of 10 saline-treated animals huddled over the pup during the 20 minute of the test. Atropine also did not affect the expression of any specific domain of alloparental behavior (p>.05), although it did significantly increase the latency to approach the pup (atropine = 45.16±8.37 sec, saline = 21.48±4.19 sec, [F(1,17) = 5.074, p = 0.038]).

In a separate pilot study we also examined the possibility that the effects of atropine on pup approach seen in Experiment 6 might have been due to a deficit in motor abilities. The same time points and dosages described in Experiment 6 were used in a group of telemetry-implanted animals tested alone in their home cage. The telemetry device provides an estimate of locomotor activity. Administration of either atropine or saline injection, administered in a counter-balanced order (n = 8 subjects) did not significantly influence locomotor activity (p>.05), suggesting that the increased latency to approach the pup observed in Experiment 6 probably was not due to neuromuscular deficits.

### Experiment 7 (Immunohistochemistry)

**Figure 4:**
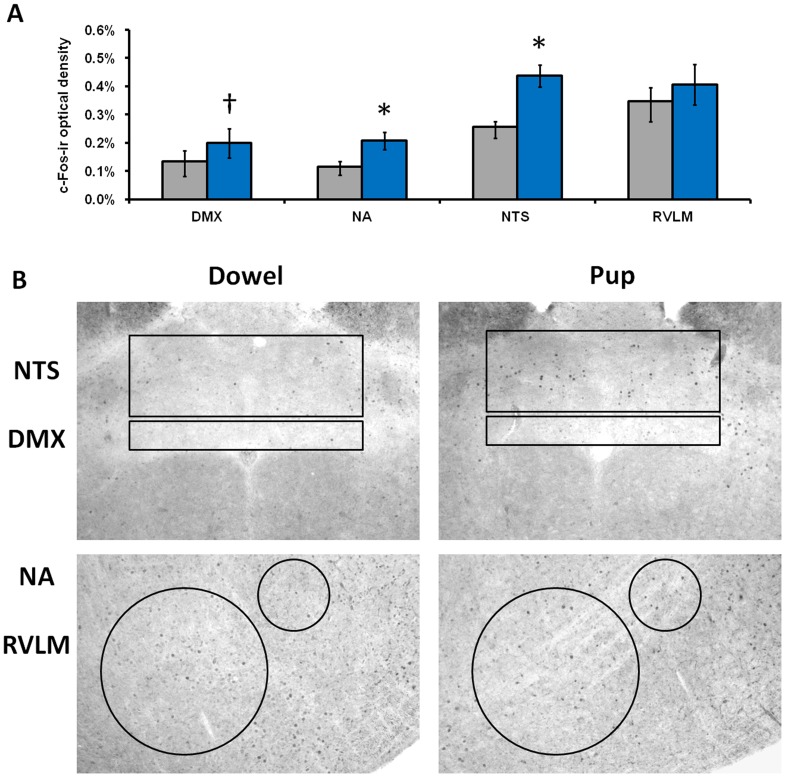
c-Fos activity is higher in brainstem autonomic nuclei related to parasympathetic function (A). Mean c-Fos optical density (+/− SEM) in the dorsal motor nucleus of the vagus (DMX), nucleus ambiguus (NA), nucleus tractus solitarius (NTS), and rostral ventrolateral medulla (RVLM) following exposure to either a dowel (gray) or pup (blue). † indicates p<0.1 * indicates p<0.05. Representative photomicrographs at 4× magnification (**B**).

Figure 4 shows brainstem c-Fos data. Animals exposed to a pup had significantly higher c-Fos-ir in the NA (p = 0.007) and the NTS (p = 0.004) compared to males exposed to a dowel. There also was a trend towards higher c-Fos-ir in the DMX in the pup condition, although this did not reach statistical significance (p = 0.069). In contrast, there was no difference in c-Fos-ir observed in the RVLM between conditions (p = 0.26). Of the 30 males exposed to a pup, 23 expressed alloparental behavior. Due to the small sample sizes and short duration of stimulus presentation in the pup attacking subset, data from the animals that did not show alloparenting were not analyzed.

## Discussion

The results of this series of experiments revealed that alloparental behavior in male prairie voles is associated with a sustained increase in heart rate. This contradicted our initial prediction that being in the presence of a pup might reduce arousal, and suggests an alternative behavioral and physiological interpretation of the emotional experiences of males during alloparental behavior.

The observed heart rate increase was specific to pup exposure. Although exposure to either another social stimulus, i.e. a novel adult female, or a wooden dowel produced a transient increase in heart rate, these stimuli did not produce the extended pattern of increased heart rate seen during pup exposure (Figure 1). Changes in heart rate could not be attributed to an increase in locomotor activity, since males with a pup were less physically active than in other conditions. The observed increase in heart rate was not simply a consequence of novelty, nor did it habituate during either repeated trials or extended 60 minute exposures to the infant. Furthermore, the body temperature of the alloparental males did not change across either a 20 minute or 60 minute pup exposure. Physical contact with the pup was essential for the increase in heart rate to occur. Previous work in the socially monogamous prairie vole led to the hypothesis that exposure to a pup was anxiolytic in adult male voles [13], [14], [17]. In contrast, the present findings suggest that pup exposure induces a state of increased sympathetic activity, possibly associated with arousal and heightened vigilance. These findings also are consistent with previous evidence that arousal or stressful experiences may increase the tendency of males to show alloparental behavior (Bales, Kim, Lewis-Reese and Carter, 2004; Bales, Kramer, Lewis-Reese and Carter, 2006).

Additionally, these results recontextualize earlier findings on the neuroendocrine elements of alloparental behavior in the vole. We previously observed a transient attenuation of plasma corticosterone following exposure to a pup [14], which was seen as potential evidence of an anxiolytic effect of pup exposure. However, when presented with a pup, males tend to show reductions in ambulation –i.e. kyphotic huddling. This sedentary action may underlie the reduction in HPA-axis activity. At the same time, our current observations suggest that cardiac sympathetic activity is high.

An infant-induced increase in heart rate is not without precedent in the literature on human infant care. Humans often respond to infant cries with an acceleration of heart rate [31]. Furthermore, in humans there is greater cardiac reactivity in non-parents than in parents and in males relative to females [32].

The increase in heart rate concomitant with alloparental behavior in male voles appears to rely on an increase in peripheral sympathetic drive to the heart. The heart rate increase during pup exposure was blocked by atenolol, a selective, peripheral β_1_ receptor antagonist, which did not affect basal heart rate or our measures of alloparental behavior, including pup approach, retrieval and arched back huddling. Atropine, in the dose used here, is known to diminish RSA by blocking the bradycardic influence of the vagus and in so doing, increases heart rate [20]. Atropine treatment increased the latency to approach the pup, although once the pup was contacted the alloparental behavior of the males did not differ from controls. In the present study atropine did not have any effects on locomotor activity. The mechanisms for the atropine-induced hesitancy to initiate approach the pup could reflect an altered perception of safety, resulting from visceral parasympathetic antagonism, or other side-effects of atropine.

The heart rate changes seen during pup exposure differ from the classic tachycardic response, in which heart rate increases are typically accompanied by vagal withdrawal. In fact, the correlation between RSA and heart period was highest in the pup condition, suggesting that heart rate was under greater vagal control [33]. Activation of both sympathetic and myelinated vagal pathways during alloparental behavior may be an example in which there is dual autonomic activation.

Increases in c-Fos in autonomic brainstem nuclei also support the conclusion that both sympathetic and parasympathetic inputs to the heart are activated during pup exposure. Exposure to a pup was associated with increased neuronal activity in the NA, which is a source nucleus for the myelinated branch of the vagus [34]. Stimulation of the NTS is known to produce activation of glutamatergic currents in the cardiac vagal neurons of the NA [35]. c-Fos activation in RVLM did not differ between groups, suggesting that both pup exposure and dowel exposure induced sympathetic activation. However, the latter conclusion is limited by the absence of an unhandled group.

Although uncommon, there are other examples of dual activation [36]. For instance, electrical stimulation of the hypothalamus, presumably through effects on the paraventricular nucleus, produced activation of both cardiac sympathetic and vagal nerves [37] and oxytocin release. Oxytocin has been implicated in social engagement and other forms of social behavior, including alloparenting in voles [17], [38]. We have previously observed that oxytocin is quickly released in male prairie voles by pup exposure, with the possibility to facilitate subsequent alloparenting [14].

The response to infant cries in humans is modulated in part by oxytocin pathways [39]–[41] and the cardiac response to infant crying varies as a function of polymorphisms in the gene for the oxytocin receptor [41]. Oxytocin may decrease the activity of neural circuitry related to anxiety during infant crying [40], but also increases sympathetic tone to the heart when acting on spinal preganglionic neurons [42]. In addition, oxytocin facilitates parasympathetic activity [43], [44] and in humans can simultaneously activate both sympathetic and parasympathetic branches of the ANS [45].

The apparent contradiction of the classic opponent process view of the ANS reinforces the need for a more refined understanding of the ANS, constructed upon an understanding of the evolution of the ANS [16]. The present findings indicate that alloparental care involves the more recently evolved, myelinated vagal input to the heart, with source nuclei in the NA. Activity in this system can occur in conjunction with increased cardiac sympathetic input. However, the features of social engagement required by alloparenting appear to be dependent on a degree of arousal (i.e., cardiac output) that cannot be accomplished by withdrawal of vagal inhibition to the heart acting alone. The somewhat novel physiological responses seen in the presence of a pup suggest that alloparenting may be an example of eustress or “good stress” [46], [47] and therefore a promising avenue for future research. However, under conditions of excessive or chronic stress, unchecked arousal could leave males vulnerable to inappropriate or aggressive reactions to the infant.

Alloparenting is an ethologically relevant behavior that can provide a window into the role of endogenous neuropeptides and their effects on the ANS. Alloparenting occurs in many mammals including primates (Hrdy, 2009). However, in humans, infant abuse or neglect are male-biased and especially common in unrelated males left to care for unrelated young [7], [8], [48]. In the present experiments conducted in male prairie voles, we observed a sustained increase in heart rate in response to caring for an infant. This heart rate increase was accomplished by an increased sympathetic drive to the heart while maintaining myelinated vagal influence. A complex cocktail of neuroendocrine processes, including oxytocin, vasopressin and CRH, may induce or support the demands of male alloparental behavior and protection of the offspring, in part through effects on the ANS. Knowledge of the psychophysiology and neuroendocrinology of parental behavior is essential to understanding the biology of healthy parenting.
